# Evaluating the Mutual Relationship between IPAT/Kaya Identity Index and ODIAC-Based GOSAT Fossil-Fuel CO_2_ Flux: Potential and Constraints in Utilizing Decomposed Variables

**DOI:** 10.3390/ijerph17165976

**Published:** 2020-08-17

**Authors:** YoungSeok Hwang, Jung-Sup Um, Stephan Schlüter

**Affiliations:** 1 Department of Climate Change, Kyungpook National University, Daegu 41566, Korea; poiu012345@naver.com; 2 Department of Geography, Kyungpook National University, Daegu 41566, Korea; jsaeom@knu.ac.kr; 3 Department of Mathematics, Natural and Economic Sciences, Ulm University of Applied Sciences, 89075 Ulm, Germany

**Keywords:** IPAT/Kaya identity, GOSAT, CO_2_ flux, correlation, hierarchical cluster analysis

## Abstract

The IPAT/Kaya identity is the most popular index used to analyze the driving forces of individual factors on CO_2_ emissions. It represents the CO_2_ emissions as a product of factors, such as the population, gross domestic product (GDP) per capita, energy intensity of the GDP, and carbon footprint of energy. In this study, we evaluated the mutual relationship of the factors of the IPAT/Kaya identity and their decomposed variables with the fossil-fuel CO_2_ flux, as measured by the Greenhouse Gases Observing Satellite (GOSAT). We built two regression models to explain this flux; one using the IPAT/Kaya identity factors as the explanatory variables and the other one using their decomposed factors. The factors of the IPAT/Kaya identity have less explanatory power than their decomposed variables and comparably low correlation with the fossil-fuel CO_2_ flux. However, the model using the decomposed variables shows significant multicollinearity. We performed a multivariate cluster analysis for further investigating the benefits of using the decomposed variables instead of the original factors. The results of the cluster analysis showed that except for the M factor, the IPAT/Kaya identity factors are inadequate for explaining the variations in the fossil-fuel CO_2_ flux, whereas the decomposed variables produce reasonable clusters that can help identify the relevant drivers of this flux.

## 1. Introduction

IPAT/Kaya identity is used to analyze the input factors of CO_2_ emissions. The IPAT identity estimates the human impact on the environment, and the Kaya identity represents the CO_2_ emissions as the product of five factors such as, for example, the gross domestic product (GDP) and population. It plays a crucial role in the construction of preliminary and future emission scenarios [1]. In addition to its simplicity, IPAT/Kaya identity is very useful to find the most effective and critical criteria for implementing carbon dioxide (CO_2_) emission reduction targets as it identifies the driving forces with regard to CO_2_ emissions from anthropogenic activities [2,3]. According to the United Nations Framework Convention on Climate Change (A/AC.237/18 (Part II)/Add.1 and Corr.1), CO_2_ emissions can be defined as the release of CO_2_ and their precursors into the atmosphere over a specified area and period of time. CO_2_ emissions can be expressed in terms of either inventory measurements or flux. The IPAT/Kaya identity uses the inventory CO_2_ emissions as environmental impacts. Inventory CO_2_ emissions present the quantity of CO_2_ estimated indirectly using the emission factors in units of weight. They contain information about CO_2_ emitted into the atmosphere by an individual, an organization, a process, a product, or an event from within the boundaries of a specific country [4]. Inventory CO_2_ emissions fluctuate depending on a variety of variables, such as the collection and reporting system of the country’s energy statistics, data definition and data processing, level of detail, and specific local conditions. Besides, accuracy, transparency, and uncertainty of inventory CO_2_ emission data vary among countries owing to the differences in proficiency and level of development of statistics [5]. Thus, documented inventory CO_2_ emissions sometimes show large discrepancy with the actual CO_2_ directly emitted to the atmosphere [6,7].

### 1.1. Benefits of IPAT/Kaya Identity

In previous studies, the (linear) correlation between all five factors of IPAT/Kaya identity and inventory CO_2_ emissions was empirically established and examined on a global scale [8]. At face value, IPAT/Kaya identity suggests that inventory CO_2_ emissions grow linearly with the increases in these factors. However, the driving forces of IPAT/Kaya identity are often not instructive because of the great heterogeneity among countries. Examples are differences in demographics, economics, resources, and technology with respect to inventory CO_2_ emissions [1,9]. A global aggregated correlation analysis between the inventory CO_2_ emissions and IPAT/Kaya identity isolates the spatial and temporal heterogeneity, particularly with respect to the distinctions between industrial and developing countries [10]. Therefore, to ascertain the true driving forces of IPAT/Kaya identity for actual CO_2_ emissions, the correlation between IPAT/Kaya identity and the standardized CO_2_ directly emitted to the atmosphere on a regional scale should be evaluated.

### 1.2. The Fossil-Fuel CO_2_ Flux

The CO_2_ flux represents the transfers of CO_2_ among different reservoirs of CO_2_ [11]. An example is the combustion of fossil fuel: the fossil CO_2_ flux indicates the amount of CO_2_ transferred from one reservoir (fossil fuel) to another (atmosphere). CO_2_ fluxes are usually expressed as a rate, that is, as an amount of substance being transferred over a certain period of time in a certain area; in this case the unit would be kgC km^2^ year^−1^. Thus, the fossil-fuel CO_2_ flux indicates the intensity of CO_2_ directly emitted to the atmosphere in standardized units. Unlike inventory CO_2_ emissions, the fossil-fuel CO_2_ flux is the footprint and absolute data. It is a measure of the direct CO_2_ emissions from CO_2_ sources on the ground to the atmosphere. Thus, this flux is objective with regard to the heterogeneity of populations and environments of individual countries and considers only the existence and locations of CO_2_ sources related to fossil-fuel combustion in a country.

### 1.3. Characteristics of the GOSAT Fossil-Fuel CO_2_ Flux

In the inversion frameworks, the fossil-fuel emissions are the most important reference for analyzing the carbon budget among the three CO_2_ fluxes, namely, the biospheric and oceanic fluxes and the fossil-fuel flux. The fossil-fuel emissions are given as known quantities, and these values cannot be corrected via optimization because fossil fuel emissions are already measured on the basis of the survey [12]. In this regard, literature suggests the application of satellite-observed CO_2_ data that have a denser spatial coverage. Emission inventory with high spatiotemporal resolution is essential for accurate inversion. The inventory CO_2_ emission data in national inventory reports (NIRs) contain the net CO_2_ emission data only within a given national boundary. These data are not sufficient to calculate regional fluxes. In contrast, satellite-based fossil-fuel CO_2_ flux data contain large amounts of information on near-ground CO_2_ sources. The Japan Aerospace Exploration Agency Greenhouse Gases Observing Satellite (GOSAT) fossil-fuel CO_2_ flux has high spatial resolution and employs the Carbon Monitoring for Action (CARMA), which is a global database of CO_2_ emissions from power plants and nighttime satellite imagery. The GOSAT fossil-fuel CO_2_ flux provides CO_2_ emissions in terms of locations of CO_2_ sources and provides a measure of the direct exchange of CO_2_ between in situ CO_2_ sources and the atmosphere over CO_2_ sources located within an area. [13,14]. Thereby, the satellite-based fossil-fuel CO_2_ flux has the advantage of monitoring and comparing the average flux from the CO_2_ sources located in heterogeneous countries because the satellite measures the CO_2_ flux all over the world with the same standardized method and unit. Evaluating mutual relationships between the factors of IPAT/Kaya identity and the satellite fossil-fuel CO_2_ flux can offer tangible evidence to validate the actual driving forces of these factors with regard to the CO_2_ directly emitted to atmosphere. Thus, the CO_2_ fossil flux is a simple, robust diagnostic property of the CO_2_ directly emitted to the atmosphere. It can provide an independent validation reference to evaluate the mutual correlation between the IPAT/Kaya identity and the CO_2_ directly emitted into the atmosphere from ground CO_2_ sources [15,16].

### 1.4. Scope of this Paper

Nonetheless, the mutual correlation of the fossil-fuel CO_2_ flux with IPAT/Kaya identity is yet to be validated. Raupach et al. [17] used the extended form of Kaya identity based on the airborne fraction of CO_2_ to assess the relative effects of changes in the airborne fractions and anthropogenic drivers of CO_2_ emissions on CO_2_ growth. They concluded that the growth of per capita income and the decline in negative growth in the carbon intensity of the economy is greatly responsible for the accelerated growth (post 2000) in the airborne fraction of CO_2_. Zhang et al. [18] demonstrated the influence of subannual variations in fossil-fuel CO_2_ emissions, which were estimated using the Kaya identity and used as the flux boundary condition, on simulated CO_2_ concentration and suggested that inversion studies should consider these variations in the affected regions. Garrett [19] remarked about the substantially narrowed visions of future emission scenarios for implementation in global circulation models, which provide projections for future climate warming based on the evolution of the factors of the Kaya identity, from a thermodynamic perspective. However, literature on the analysis of the mutual correlation between the IPAT/Kaya identity and the CO_2_ fossil-fuel flux is lacking. This study addresses this lacuna in research. Our objective is to identify the realistic driving forces of IPAT/Kaya identity on the actual CO_2_ emitted to the atmosphere.

## 2. Materials and Methods

### 2.1. Study Area

The certainties and accuracy of the energy consumption data and CO_2_ emission data in NIRs are relatively high for the countries listed in Annex 1 of the United Nations Framework Convention on Climate Change (UNFCCC) due to their well-developed statistical systems and capacity to use higher-tier methods [20]. Europe is the second-smallest continent in the world after Australia. As 44 countries are densely located in this region, it is an ideal region for studying carbon emissions among countries. Europe is also ideal for investigating the correlation between fossil-fuel CO_2_ flux and IPAT/Kaya identity owing to the diversity in structure of the energy consumption, population, industry, and economic scale [21]. To guarantee the accuracy of fossil-fuel CO_2_ flux data, a high accuracy of preliminary data for CO_2_ emission is required. Analysis of the sum of Annex I reported emissions as well as some independent estimates and inverse modeling results found an uncertainty of 6 percent for fossil-fuel CO_2_ [20]. In this regard, GOSAT fossil-fuel CO_2_ flux is calibrated with accurate CO_2_ emissions data. From the list of Annex 1 countries, we selected 30 European countries excluding the smallest and most remote ones. For example, we excluded Iceland, Monaco, Liechtenstein, and Malta because these countries are either too far from the European continent or too small for using 1° × 1° scale GOSAT fossil-fuel CO_2_ flux data.

### 2.2. IPAT/Kaya Identity

#### 2.2.1. Description of IPAT/Kaya Identity

The IPAT identity is widely used to examine the drivers of CO_2_ emissions. The identity (I = P × A × T) states that the human impact on the environment (I) is the product of population (P), affluence (A), and technology (T). As shown in Equation (1), the Kaya identity distinguishes the factors of P, A, and T with respect to CO_2_ emissions into four: (1) the size of the population, (2) GDP per capita, (3) energy intensity of the GDP, and (4) carbon footprint of energy [1,22]. In Equation (1) we additionally split up the factor T into energy divided by the GDP and CO_2_ divided by energy.

(1) $$CO_{2}\;\;emissions\;=Population\;\times \frac{GDP}{Population}\times \frac{Energy}{GDP}\times \frac{CO_{2\;}emissions}{Energy}\;\;\;\;\;$$

Using Equation (1), many studies extended the IPAT identity to the Kaya identity to explore the energy sector in detail, as explained in Equation (2) [22,23,24,25]:

(2) $$\begin{array}{ll} CO_{2}\;emissions\; & =Population\;\times \frac{GDP}{Population}\times \frac{TEC}{GDP}\times \frac{EC}{TEC}\times \frac{CO_{2\;}emissions}{EC}\;\\ & =\;P\times G\times I\times M\times E, \end{array}$$

where P is the population size; GDP, the gross domestic product; TEC, the total energy consumption; and EC, the fossil fuel energy consumption. In this equation, E (CO_2_/EC) is the CO_2_ emission coefficient related to fuel sources; M (EC/TEC), the portion of fossil-fuel consumption from the total energy consumption; I (TEC/GDP), the energy intensity; G (GDP/P), per capita GDP; and P, population size [22]. In this study, we used the Kaya identity as described in Equation (2).

#### 2.2.2. Data Sets for Computing the Decomposed Variables of IPAT/Kaya Identity

The International Energy Agency (IEA) collects energy supply and demand data not only for the member countries of the Organization for Economic Cooperation and Development (OECD), but also for non-OECD countries [26,27]. The original data are submitted by national administrations of the OECD, European Union (EU), and United Nations Economic Commission for Europe (UNECE) member states. The (final) joint IEA/OECD–Eurostat–UNECE questionnaire is the result of aggregating a set of five individual questionnaires (for coal, oil, gas, electricity, and renewable energy) [28]. Then, the basic energy statistics with over 60 energy types in physical energy units such as ton and m^3^ are converted into energy units (ktoe). This disaggregated energy balance is combined into 13 energy types (coal, crude oil, biofuels, nuclear, etc.). The sum of these 13 energy types gives the total energy consumption [29,30]. We used the total final consumption sections from the IEA energy balance data for TEC and the EC to calculate the I, M, and E factors of the Kaya identity from 2010 to 2017 [31]. The GDP and population data were acquired from World Bank data to calculate G, I, and P in the IPAT/Kaya identity from 2010 to 2017 [32].

An NIR contains detailed qualitative and quantitative information and tables in a common reporting format (CRF) for all Kyoto Protocol, such as carbon monoxide (CO), nitrogen oxides (NO_x_), non-methane volatile organic compounds, and sulfur dioxide (SO_2_) [33,34]. The GOSAT Level 4a fossil-fuel CO_2_ flux exclusively provides the annual CO_2_ flux derived from fossil-fuel combustions with 1° × 1° spatial resolution. It is generally acknowledged that CO_2_ accounts for the most significant portion of greenhouse gases, and the term CO_2_ is often used interchangeably with greenhouse gas. To perform a correlation analysis between factors in the Kaya identity and GOSAT fossil-fuel CO_2_ flux data, we used direct CO_2_ emissions (CRF Table 10s2 submitted to UNFCCC in 2018) from 2010 to 2017. We excluded the CO_2_ emissions from land use, land use change, and forestry sectors since they are associated with the variations in CO_2_ uptakes and emissions from the net CO_2_ sink (i.e., forests).

The a priori flux dataset for the GOSAT fossil-fuel CO_2_ flux data inversion comprises monthly fossil-fuel CO_2_ emissions with the Open-source Data Inventory of Anthropogenic CO_2_ emissions (ODIAC). ODIAC data are obtained by merging the CARMA database, nighttime satellite imagery, and the Carbon Dioxide Information Analysis Center (CDIAC) datasets [35,36]. The ODIAC inventory dataset describes precisely the local spatial structures of large cities by using nighttime data. The ODIAC dataset can depict the spatial variability in CO_2_ emission levels even in city centers with the standard measurements from the Defense Meteorological Program—Operational Line-Scan System (DMSP—OLS) instruments. Thus, a complete picture of the fossil-fuel emissions for the GOSAT fossil-fuel CO_2_ flux is obtained [37]. The ODIAC-based GOSAT fossil-fuel CO_2_ flux provides improved spatial distribution of fossil-fuel CO_2_ emissions because of the large point-source data and nighttime observations employed.

In order to compare the factors’ fluctuations, we computed the coefficient of variation (CV), which is the result of dividing the standard deviation of a data set by its mean. Hence, the CV indicates the variation in relation to the average level of the respective factor. As displayed in Table 1, we see that the standard deviations of both the P factor and population are larger than those of other factors. However, considering the CV values, EC and TEC show the highest values. This is reasonable as, in this study, we used data from 30 quite heterogeneous European countries. For examples, the Netherlands (1.74 ktoe/km^2^) have the 29 times larger TEC than Latvia (0.06 ktoe/km^2^).

### 2.3. Multiple Regression and Cluster Analysis

To evaluate the mutual dependencies in our data sets, that is, the dependencies between the factors of the IPAT/Kaya identity (or the corresponding decomposed variables of these factors), we established a regression of the GOSAT fossil-fuel CO_2_ flux above-mentioned factors. The corresponding multiple regression models are shown in Equations (3) and (4). They were calibrated using ordinary least squares optimization:

*Fossil-fuel CO_2_ flux* = α_0_ + α_1_ × *P* + α_2_ × *G* + α_3_ × *I* + α_4_ × *M* + α_5_ × *E* + *ε*_1*,*_ (3)

*Fossil-fuelCO*_2_*flux* = *β*_0_ + *β*_1_ × *Population* + *β*_2_ × *GDP* + *β*_3_ × *TEC* + *β*_4_ × *EC* + *β*_5_ × *CO*_2_*emissions* + *ε*_2_ (4)

where $\alpha_{i},\beta_{j}\in \mathbb{R},\;i,j=1,\ldots ,5$, and $\epsilon_{1}$ and $\epsilon_{2}$ are Gaussian distributions with zero mean and standard deviation σ>0. Equations (3) and (4) show the regression models based on the five factors and the decomposed variables of the factors in the IPAT/Kaya identity. The pairs of data per country are eight years’ data from 2010 to 2017 and we used 240 samples per individual variable. Note that, in order to be able to merge the datasets of these eight individual years to one large sample, we had to demand the absence of autocorrelation. With autocorrelation we mean (partial) dependence of a data set on its own past, that is, there is a correlation on the time axis. For this purpose, we applied the Durbin–Watson test. Autocorrelation might also occur if the functional form of the model itself is incorrect. The Durbin–Watson statistic is an indicator of autocorrelation in the residuals of a regression model: values greater than 0 but less than 2.0 indicate positive correlation; values close to 2.0 indicate no autocorrelation; values from 2 to 4 indicate negative autocorrelation [38]. For the regression models in Equations (3) and (4), the values of the test statistic are 2.13 and 2.07, respectively, and hence, both satisfy the assumptions regarding the autocorrelation of the error term. Hence, we can fit both models from Equations (3) and (4) using the merged dataset. Thereby, the decomposed variables are fitted to annual net amounts, and the fossil-fuel CO_2_ flux is fitted to annual mean values.

Cluster analysis is an exploratory approach that intends to identify structures within a dataset by segmenting it into disjoint sub-groups of similar (possibly multivariate) observations. Cluster analysis methods can be applied to binary, nominal, ordinal, and scale (interval or ratio) data. Some of the commonly used methods are hierarchical clustering, k-means, clustering large applications (CLARA), or the Ward algorithm [39]. Thereby, cluster analysis is often used in conjunction with other methods such as discriminant analysis. After clustering, the members within a group should have similar properties and features, while those in different groups should have highly dissimilar properties and features. This is achieved using certain distance measures. For example, in Ward’s method, a hierarchical approach, analysis of variance is performed to evaluate the distances between the cluster centroids; this method optimizes the minimum variance within clusters by using the sum of squared deviations within the individual groups to evaluate cluster membership. Thereby, a meaningful data structure can be applied to various types of data without prior information about the internal structure of the dataset.

Note that a clustering algorithm does not distinguish between dependent and independent variables. Hence, to use it in our study, we applied Ward’s method to multivariate observations obtained by combining the country-specific fossil-fuel CO_2_ flux value with the input factors, that is, the independent variables from Equations (1) and (2). We obtained various sets of multivariate observations and performed a cluster analysis for each variable to explore the unknown patterns and characteristics of both dependent and independent variables that influence the results of the multiple regressions from Equations (1) and (2). If these variables have high positive correlation, the different groups will be linearly located on the trend lines with distinctive range between different groups. Thus, by performing a cluster analysis, we obtained more information about the structures and characteristics of different groups of independent variables (i.e., the factors of IPAT/Kaya identity and the decomposed variables of IPAT/Kaya identity) and their influence on the dependent variable, that is, the fossil-fuel CO_2_ flux.

## 3. Model Estimation and Evaluation of Results

We employed the methods described in Section 2.3. and the variables derived in Section 2.2.: we fit the multiple regression models from Equations (3) and (4) and applied Ward’s clustering.

### 3.1. Model Calibration

The data in Table 2 show that the multiple regression model from Equation (3) has a relatively low explanatory power with an R² of 0.38. Correlation and regression coefficient values are also relatively low, except for the M factor. From the *p*-values of the regression coefficients, we see that among the five factors of the Kaya identity (hereinafter, Decomposition 1), the coefficients of I and E are statistically insignificant. Hence, their influence on the fossil-fuel CO_2_ flux cannot be proven using the model in Equation (3). This is remarkable as the E factor, that is, the CO_2_ emissions from fossil fuel/EC, was expected to be strongly and positively related to the fossil-fuel CO_2_ flux. However, the *p*-value indicates the factor’s insignificance, and the correlation coefficient shows only a small negative influence.

In contrast, we see that the explanatory power of the multiple regression model based on the decomposed variables of IPAT/Kaya identity factors (hereinafter, Decomposition 2) is comparatively high (R^2^ = 0.83). In addition, the correlation coefficients between Decomposition 2 and the fossil-fuel CO_2_ flux (0.64 to 0.90) are higher than the corresponding values of Decomposition 1 (−0.23 to 0.56). The results for all the models are listed in Table 3. Interestingly, some factors of the IPAT/Kaya identity and their decomposed variables differ in terms of the significance (*p*-value) of their regression models. For example, the E factor in the model based on Equation (3) is insignificant with a *p*-value of 0.58. However, its decomposed variables, namely, EC and CO_2_ emissions from fossil fuels, individually are significant (*p* ≤ 0.01) in the model from Equation (4). The I factor is insignificant (*p* = 0.66) in the first regression model, but the GDP, which is a component of the I factor, is significant (*p* ≤ 0.01) in the second model. The TEC value, again, which is another decomposed variable of the I factor, is statistically insignificant due to its large *p*-value of 0.66. It is the only insignificant variable in the second model. This finding suggests that changes in the TEC are not associated with changes in the response of the fossil-fuel CO_2_ flux. An explanation may be that TEC contains the energy consumptions from 13 energy types, from fossil fuels to nuclear and renewable energies, all converted to the energy units (ktoe). Hence, the proportion of nonfossil fuels accounts for about 20% (Netherlands) to 67% (Sweden) in TEC. However, as fossil fuel accounts for over 50% in the energy mix of all countries except for Estonia, Finland, Latvia, Norway, and Sweden (from our data for 2010–2017), we still see a fairly positive correlation of 0.64 with the fossil-fuel CO_2_ flux. This example proves that computing the correlation is often not enough, and additional insight is gained by calibrating the model in Equation (4).

### 3.2. Using Cluster Analysis to Handle the Problem of Multicollinearity

Despite this knowledge gained by calibrating the model in Equation (4), a challenge persists: we see significant multicollinearity, that is, the decomposed factors of the IPAT/Kaya identity are not independent of each other. This fact has been well established in previous research [1,40,41]. We used the variance inflation factors (VIFs) as indicators of multicollinearity. The general rule of thumb is that VIFs > 4 warrant further investigation, while VIFs > 10 are signs of serious multicollinearity requiring correction [3]. In the first model, the VIF values are all far below 10, whereas in the second model, three out of five factors exceed 10. Multicollinearity is commonly observed along with high R², as observed in Table 3. Besides, when analyzing the correlation between the individual factors of Decomposition 2, we see some substantial interdependencies between the factors TEC and EC or between the population and GDP (Table 4). These interdependencies make it difficult to interpret the results given in Table 3. Hence, further analysis is required to support our deductions.

**Figure 1:**
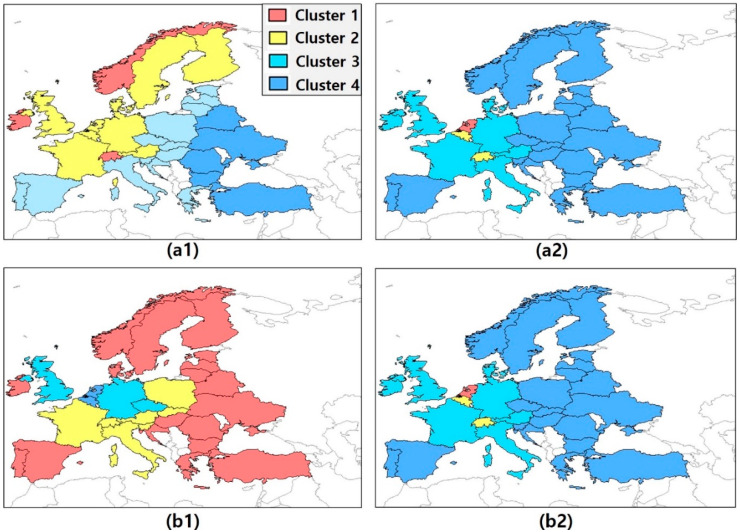
Distribution map of country clusters on the basis of fossil-fuel CO_2_ flux, Kaya identity (G and I factors), and decomposed variables of G and I factor (GDP, population, TEC). (**a1**) Country cluster with the fossil-fuel CO_2_ flux and G factor. (**a2**) Country cluster with the fossil-fuel CO_2_ flux and decomposed variables of G factor (GDP and population). (**b1**) Country cluster with the fossil-fuel CO_2_ flux and I factor. (**b2**) Country cluster with the fossil-fuel CO_2_ flux and decomposed variables of I factor (GDP and TEC).

**Figure 2:**
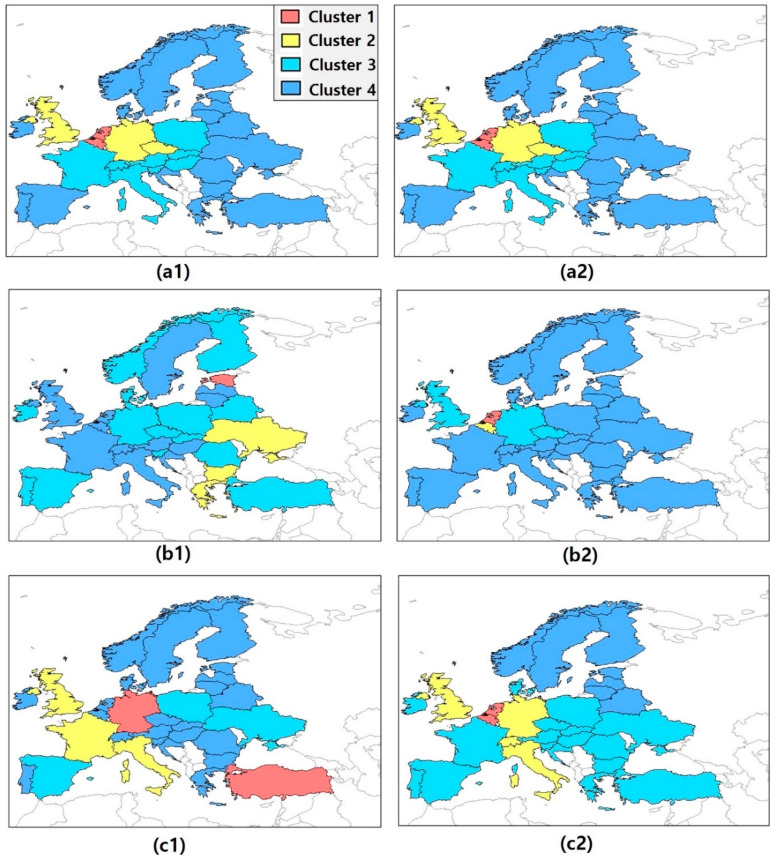
Distribution map of country clusters on the basis of the fossil-fuel CO_2_ flux, Kaya identity (M, E, and P factors), and decomposed variables of M, E, and P factors (EC, TEC, CO_2_ emissions from fossil fuel, and population). (**a1**) Country cluster with the fossil-fuel CO_2_ flux and M factor. (**a2**) Country cluster with the fossil-fuel CO_2_ flux and decomposed variables of M factor (EC, and TEC). (**b1**) Country cluster with the fossil-fuel CO_2_ flux and E factor. (**b2**) Country cluster with the fossil-fuel CO_2_ flux and decomposed variables of E factor (CO_2_ emissions from fossil fuel and EC). (**c1**) Country cluster with the fossil-fuel CO_2_ flux and P factor. (**c2**) Country cluster with the fossil-fuel CO_2_ flux and decomposed variables of P factor (population).

For this purpose, we performed a multivariate cluster analysis for both Decomposition 1 and 2. The individual cluster pattern was derived based on a bivariate dataset (over all 30 countries and years) consisting of the fossil-fuel CO_2_ flux on the one side and one of the five factors of the IPAT/Kaya identity or their decomposed factors on the other side. Then, we could explore the disparity of the results in Table 2 and Table 3 without assuming a specific model. The property of multicollinearity is also observed in the results of the cluster analysis performed using Ward’s method (see Section 2.3.). Details of the clustering are provided in Table A1 and Table A2, but the major results can be also obtained by examining Figure 1 and Figure 2, which show colored maps to visualize the clustering. In Figure 1a1,b1, we show the resulting clusters for G and I, respectively, while in Figure 1a2,b2, we show the resulting clusters for the corresponding decomposed variables. Each color represents a different cluster.

The maps on the left side show no clear structure or specific pattern, whereas the maps on the right side indicate (strong) positive correlation, and we see reasonable clusters such as Central Europe and Eastern Europe. Let us, for example, consider the clusters based on the factors G and I (for details, see Table A1). Norway, Ireland, and Switzerland belong to the first cluster with the highest G factor values among the sample (0.07–0.08 MM $/person). However, the corresponding fossil-fuel CO_2_ flux values are not the highest in the sample (which would indicate a positive dependence). Besides, considering again the G factor-based clustering, the fossil-fuel CO_2_ flux values in Cluster 1 show a considerably large range (0.08–0.70 gC m^2^ day^−1^) which fully contains all values of Cluster 4 (0.25–0.37 gC m^2^ day^−1^). Cluster 2, again, has a considerably large range of fossil-fuel CO_2_ flux values comprising the smallest and the largest values (0.05–3.80 gC m^2^ day^−1^). Hence, we cannot derive any relation between the G factor and the fossil-fuel CO_2_ flux from this clustering result. Considering the decomposed factors of the G factor, again we see more evidence for a relation. The Netherlands show the highest values for GDP (24.66 MM $/km^2^), population (507.89 person/km^2^), and fossil-fuel CO_2_ flux (2.48 gC m^2^ day^−1^). With decreasing GDP, we also have tendentially a decreasing fossil-fuel CO_2_ flux, whereby there is a certain range in each cluster. Looking at the I factor, we have an (on average) increasing pattern of fossil-fuel CO_2_ flux values from Cluster 1 (0.05–0.49 gC m^2^ day^−1^) to Cluster 4 (2.48–3.80 gC m^2^ day^−1^). The I factor values are slightly decreasing, whereas the ranges of values within the individual cluster are fairly large and overlap each other. As a consequence, we can hardly see any interdependency between the I factor and the fossil-fuel CO_2_ flux.

Similar conclusions can be drawn by analyzing Figure 2a1–c1, where we compare the clusters for M, E, and P with the clusters of their decomposed variables (for details see Table A2). The ranges of M factor values in all clusters overlap more or less, whereas the fossil-fuel CO_2_ flux values are decreasing. E factor values, again, are decreasing where ranges of the cluster values hardly overlap. However, the corresponding fossil-fuel CO_2_ flux values do. The fossil-fuel CO_2_ flux values of Cluster 3 range from 0.08 gC m^2^ day^−1^ to 1.73 gC m^2^ day^−1^, which completely includes the range of Cluster 2 (0.25–0.36 gC m^2^ day^−1^). In addition, considering the E factor, Germany, Norway, and Finland are in the same group as Turkey, Belarus, and Romania, which have a lower efficiency of generating electricity and where coal-fired power plants are dominant (Figure 2b1). The corresponding decomposed values offer a more concrete clustering, however the fossil-fuel CO_2_ flux value increases from the first to the second cluster. Apart from that, all input values as well as fossil flux values decrease, hence we see a clear positive relationship. Most of the clusters identified based on the individual decomposed variables show a distinctive pattern with quite homogeneous groups and fairly large distances between the individual clusters. This reflects the heterogeneous characteristics of the individual countries in Europe. Belgium, for example, always belongs to Cluster 1, in which the decomposed variables and the fossil-fuel CO_2_ flux show the highest values among all four clusters; Germany and UK are always in the same cluster. Northern and eastern European countries usually belong to the same Cluster as well (Figure 1a2,b2, Figure 2a2,c2).

Thus, the results of the cluster analysis indicate that Decomposition 2 has a stronger explanatory power for the fossil-fuel CO_2_ flux than Decomposition 1. Besides, except for the M factor and its decomposed variables, the cluster results on the left side of Figure 1 and Figure 2 differ significantly from those on the right side (which are based on the decomposed variables). Note that the M factor is a proportional factor that indicates the share of fossil fuels in total energy consumption [22]. Unlike other factors of the IPAT/Kaya identity, the M factor has the same cluster members in both models, that is, the model considering the correlation of M with the fossil-fuel CO_2_ flux and that considering the correlation between its decomposed variables (EC and TEC) and the fossil-fuel CO_2_ flux.

### 3.3. Discussion

Consumption-based CO_2_ emissions differ from conventional production-based inventories due to imports and exports of goods and services that entail CO_2_ emissions either directly or indirectly. However, the CO_2_ emissions in the Kaya identity account for only those CO_2_ emissions produced within national boundaries. It does not consider the CO_2_ emissions conveyed through international trade. For instance, if oil is imported for electricity generation, this results in an increase in emissions in the importing country. Whereas, if electricity as such is imported, it is not counted as emissions in the importing country. In countries like Switzerland, Sweden, Austria, the United Kingdom, or France, over 30% of consumption-based emissions were imported, with net imports to many Europeans of over 4 tons of CO_2_ per person in 2004 [42]. TEC includes the imported energy from other countries. European countries may have a low production of electricity but consume much more electricity that was produced elsewhere (leading to a higher carbon footprint). Thus, the direct CO_2_ emissions may not agree well with the energy consumed. In this study, we do not involve the net effect of CO_2_ emissions embodied in trade. This incongruence is not discussed in the current manuscript.

Besides, in the EU, 71% of the total energy is consumed by the end users. Transformation and distribution losses account for 24% of the EU’s primary energy and about 5% by the energy sector’s own consumption of energy. A 2% increase of transformation efficiency in traditional power plants, given the same fuel mix, would save about 50 million tons of CO_2_ emissions per year in the EU [43]. In this regard, we need further research about the driving forces of Kaya identity factors and decomposed variables according to variations of efficiency in energy transformation and distribution.

## 4. Potential and Constraints in Utilizing Decomposed Variables

The hypothesis of the IPAT/Kaya identity is that its five factors can be used to discuss the primary driving forces of inventory CO_2_ emissions [40]. However, in reality, these five factors are often not instructive for discussing the primary driving forces on the CO_2_ directly emitted to the atmosphere. Alternatively, as described in Section 3, the five factors of IPAT/Kaya identity can be decomposed into five subcomponents, GDP, TEC, EC, CO_2_ emissions from fossil fuel, and population. However, the multiple regression model based on these five subcomponents shows significant multicollinearity. This limits the application of this model. Owing to the interdependencies among the decomposed variables, the influence of each decomposed variable on the fossil-fuel CO_2_ flux may be overestimated. A further drawback of the five decomposed variables is that, unlike the identity factors themselves, the decomposed variables are not adequate for prioritizing targets to mitigate the domestic CO_2_ emission [1]. The decomposed variables cannot be used to identify specific categories of anthropogenic activities such as social, economic, industrial, and biophysical activities. For instance, GDP and TEC themselves do not provide any insights about the “targets” for reducing CO_2_ emissions because the absolute numbers of both decomposed variables depend on population, economic scales, energy mix, industrial structures, and so forth. Thus, the decomposed variables of IPAT/Kaya identity are not adequately indicative of the major sectors that may help to mitigate the resulting CO_2_ emissions in individual countries [44].

On the other hand, the decomposed variables have the advantage that they can be used to explain and describe the heterogeneous country-specific characteristics and levels of CO_2_-emitting activities. The decomposed variables contain instructive information about the anthropogenic CO_2_-emitting activities. Many authors, for example, demonstrated that atmospheric CO_2_ concentrations grow linearly with the five decomposed variables (GDP, TEC, EC, population, and CO_2_ emissions from fossil fuel) [45,46,47]. They are the representative parameters related to the direct CO_2_ emitted from anthropogenic activities. Thus, the decomposed variables facilitate a comparison of the intensities of CO_2_-emitting anthropogenic activities from individual countries.

As described, the IPAT/Kaya identity is a concept of splitting up the inventory CO_2_ emissions into five factors. Hence, the growth rates of the components are additive, that is, the total growth rate of the inventory CO_2_ emissions related to energy is the sum of the growth rates of the individual factors. When predicting future CO_2_ emissions, the inferred growth rates of the individual IPAT/Kaya identity factors serve as input for predicting future CO_2_ emissions or designing various emission scenarios. Thus, one of the important caveats of applying IPAT/Kaya to emission scenarios is that the five factors of IPAT/Kaya identity on the right side of Equation (2) should not be considered as the fundamental driving forces themselves [1]. The IPAT/Kaya identity assumes that each factor has the same importance in explaining the driving forces behind inventory CO_2_ emissions. The fossil-fuel CO_2_ flux is a footprint originating from the same CO_2_ sources as in the inventory CO_2_ emissions. Thus, the five factors of the IPAT/Kaya identity should be positively correlated with this flux. However, in this study, all factors of the IPAT/Kaya identity, except for the M factor, show low correlation with the fossil-fuel CO_2_ flux. In contrast, four out of five decomposed variables show a high correlation with the flux. According to this study, variations in the individual IPAT/Kaya identity factors do not always positively lead to changes in the CO_2_ directly emitted to the atmosphere. The IPAT/Kaya identity factors are calculated by dividing two specific decomposed variables. This calculation process eliminates the multicollinearity among the decomposed variables and reduces the influences of the decomposed variables on the fossil-fuel CO_2_ flux. Ignoring the correlations of the decomposed variables with this flux during the construction of the emission scenarios may lead to incorrect predictions of the actual CO_2_ emissions, which reflect the variations in the CO_2_ directly emitted to the atmosphere. Therefore, the correlation coefficients of the decomposed variables must be considered when building CO_2_ emission scenarios in order to find realistic reduction targets for atmospheric CO_2_.

## 5. Conclusions

The evaluation of the mutual correlation between the factors of the IPAT/Kaya identity and their decomposed variables with the fossil-fuel CO_2_ flux, which is the CO_2_ emitted from the in situ fossil-fuel CO_2_ sources to the atmosphere, showed disparity between the two datasets. The decomposed variables of the IPAT/Kaya identity have a substantially higher correlation with this flux than the factors of the IPAT/Kaya identity. In addition, the individual factors of the IPAT/Kaya identity are statistically insignificant when used in a regression model to explain this flux. In contrast, the decomposed variables of the IPAT/Kaya identity are statistically significant, but show multicollinearity, when considering their regression to explain the fossil-fuel CO_2_ flux. These results show that the influences and multicollinearity of individual decomposed variables on actual CO_2_ emissions are not reflected in the factors of the IPAT/Kaya identity and multiplicative calculations. However, the factors of IPAT/Kaya identity are still important for policymakers since the decomposed variables cannot provide policy-wise targets for reducing CO_2_ emissions at the national level. There are limitations to generalizing the results of this study owing to the relatively short period of 8 years of (annual) observations and the confined study area. Therefore, further research with a longer period of observations and worldwide data is necessary to generalize the results of this study. In particular, a longer period of 20–25 years (or even longer if possible) should be considered for the generalization of the results; many similar studies have considered such longer periods.

## Figures and Tables

**Table 1:** Descriptive statistics for the Greenhouse Gases Observing Satellite (GOSAT) Level 4a gridded fossil-fuel CO_2_ flux, five variables (G, I, M, E, and P) of the Kaya identity and the decomposed variables of five variables in Kaya identity of 30 European countries from 2010 to 2017.

Category		Min	Max	Mean	STDEV	CV (%)
Kaya identity	G factor (MM $/person)	0.00	0.10	0.03	0.02	0.70
	I factor (ktoe/MM $)	0.03	0.56	0.11	0.09	0.81
	M factor (ktoe)	0.34	0.80	0.62	0.11	0.18
	E factor (kt CO_2_ Equation/ktoe)	0.41	14.76	5.22	2.22	0.42
	P factor (MM person)	1.32	82.66	21.68	24.50	1.13
Decomposed variables of Kaya identity	GDP (MM $/km^2^)	0.16	26.80	4.49	5.89	1.31
	Population (person/km^2^)	13.39	507.89	123.32	104.24	0.85
	TEC (ktoe/km^2^)	0.05	1.93	0.30	0.37	1.22
	EC (ktoe/km^2^)	0.02	1.54	0.21	0.29	1.40
	CO_2_ emission (kt CO_2_ Equation/km^2^)	0.10	5.40	0.88	1.03	1.17
Fossil-fuel CO_2_ flux (gC m^2^ day^−1^)		0.06	3.79	0.68	0.78	1.14

Min: Minimum, Max: Maximum, Mean: Average, CV: coefficients of variation, STDEV: Standard deviation, TEC: total energy consumption, EC: fossil fuel energy consumption.

**Table 2:** Results of the multivariate linear regression and Pearson correlation coefficients between GOSAT fossil-fuel CO_2_ flux and the five factors of the Kaya identity.

Category		Standardized Coefficient	VIF	T-Statistics	Pearson Correlation Coefficient
Kaya identity	G	0.26 **	1.80	3.84	0.18 **
	I	0.03	1.63	0.41	−0.18 **
	M	0.66 **	1.44	10.68	0.56 **
	E	0.03	1.31	0.56	−0.23 **
	Population	−0.13 *	1.29	−2.18	0.16 **

R: 0.62; R^2^: 0.38; Durbin–Watson: 2.13; F-value (*p*-value): 29.22 (0.00); *: *p* ≤ 0.05, **: *p* ≤ 0.01.

**Table 3:** Results of the multivariate linear regression and Pearson correlation coefficients between GOSAT fossil-fuel CO_2_ flux and the decomposed variables of the five factors of the Kaya identity.

Category		Standardized Coefficient	VIF	T-Statistics	Pearson Correlation Coefficient
Decomposed variables of five factors in Kaya identity	CO_2_ emission	0.30 **	12.94	3.08	0.89 **
	TEC	0.02	1.97	0.44	0.64 **
	EC	0.56 **	22.86	4.36	0.90 **
	GDP	−0.35 **	7.28	−4.89	0.77 **
	Population	0.38 **	16.08	3.55	0.87 **

R: 0.91; R^2^: 0.83; Durbin–Watson: 2.07; F-value (*p*-value): 234.05 (0.00); *: *p* ≤ 0.05, **: *p* ≤ 0.01.

**Table 4:** Correlation between the decomposed factors of the IPAT/Kaya identity.

Category	CO_2_ Emission	GDP	Population	TEC	EC
CO_2_ emission	1.000	0.325	0.037	−0.086	−0.221
GDP	-	1.000	0.430	−0.196	−0.300
population	-	-	1.000	−0.091	−0.307
TEC	-	-	-	1.000	−0.834
EC	-	-	-	-	1.000

## Abbreviations

CARMA	Carbon Monitoring for Action
CV	Coefficient of variation
DMSP-OLS	Defense Meteorological Program—Operational Line-Scan System
EC	Fossil-fuel energy consumption
E	CO_2_ emissions/EC
G	GDP/P
GDP	Gross domestic product
GOSAT	Japan Aerospace Exploration Agency Greenhouse Gases Observing Satellite
I	TEC/GDP
IEA	International Energy Agency
M	EC/TEC
NIR	National inventory report
ODIAC	Open-source Data Inventory of Anthropogenic CO_2_ emissions
OECD	Organization for Economic Cooperation and Development
P/Pop	Population size
TEC	Total energy consumptions
UNFCCC	United Nations Framework Convention on Climate Change
UNECE	United Nations Economic Commission for Europe

## Appendix A

**Table A1:** Results obtained by the Ward methods in the hierarchical cluster analysis and comparisons of the members in individual cluster groups in terms of factors of the Kaya identity (G and I factors) and the decomposed variables of the Kaya identity (GDP, population, and TEC). G factor: MM $/person, I factor: ktoe/MM $, GDP: MM $/km^2^, Pop (Population): MM person/km^2^, TEC: ktoe/km^2^, Fossil-fuel CO_2_ flux: gC m^2^ day^−1^.

Cluster Level.	Cluster 1	Cluster 2	Cluster 3	Cluster 4
Decomposed Variables of G Factor	GDP: 24.66	GDP: 16.61–17.21	GDP: 4.72–11.02	GDP: 0.27–2.80
	Pop: 507.89	Pop: 213.88–375.67	Pop: 69.78–273.05	Pop: 14.45–137.16
	Fossil-fuel CO_2_ flux:	Fossil-fuel CO_2_ flux:	Fossil-fuel CO_2_ flux:	Fossil-fuel CO_2_ flux:
	2.48	0.70–3.80	0.33–1.73	0.08–1.32
Countries Belonging to the Cluster	NET	BEL, SWI	AUS, FRA, GER, IRE, ITA, UK	BEL, BUL, CRO, CZE, EST, FIN, GRE, HUN, LAT, LIT, NOR, POL, ROM, SI, SK, SPA, SWE, TUR, UKA
G Factor	G: 0.07–0.08	G: 0.04–0.06	G: 0.01–0.03	G: 0.00–0.01
	Fossil-fuel CO_2_ flux:	Fossil-fuel CO_2_ flux:	Fossil-fuel CO_2_ flux:	Fossil-fuel CO_2_ flux:
	0.08–0.70	0.05–3.80	0.09–1.32	0.25–0.37
Countries Belonging to the Cluster	NOR, IRE, SWI	AUS, BEL, DEN, FIN, FRA, GER, NET, SWE, UK	CRO, CZE, EST, GRE, HUN, ITA, LAT, LIT, POL, POR, SI, SK, SPA	BLR, BUL, ROM, TUR, UKR
Decomposed Variables of I factor	GDP: 24.66	GDP: 16.61–17.21	GDP: 4.72–11.02	GDP: 0.27–2.80
	TEC: 1.74	TEC: 0.47–1.34	TEC: 0.16–0.65	TEC: 0.06–0.35
	Fossil-fuel CO_2_ flux:	Fossil-fuel CO_2_ flux:	Fossil-fuel CO_2_ flux:	Fossil-fuel CO_2_ flux:
	2.48	0.70–3.80	0.33–1.73	0.08–0.35
Countries Belonging to the Cluster	NET	BEL, SWI	AUS, FRA, GER, IRE, ITA, UK	BLR, BUL, CRO, CZE, EST, FIN, GRE, HUN, LAT, LIT, NOR, POL, ROM, SI, SK, SPA, SWE, TUR, UKA
I Factor	I: 0.05–0.54	I: 0.04–0.15	I: 0.06–0.13	I: 0.08–0.09
	Fossil-fuel CO_2_ flux:	Fossil-fuel CO_2_ flux:	Fossil-fuel CO_2_ flux:	Fossil-fuel CO_2_ flux:
	0.05–0.49	0.58–0.77	1.10–1.73	2.48–3.80
Countries Belonging to the Cluster	BEL, BUL, CRO, DEN, EST, FIN, GRE, HUN, IRE, LAT, LIT, NOR, POR, ROM, SPA, SWE, TUR, UKR	AUS, FRA, ITA, POL, SI, SK, SWI	CZE, GER, UK	BEL, NET

**Table A2:** Results obtained by the Ward methods in the hierarchical cluster analysis and comparisons of the members in individual clusters in terms of the factors of the Kaya identity (M, E, and P factors) and decomposed variables of the Kaya identity (GDP, population, TEC, EC, and CO_2_ emission from fossil fuel). M factor: ktoe, E factor: kt CO_2_ Equation/ktoe, P factor: MM person, GDP: MM $/km^2^, Pop (Population): MM person/km^2^, TEC: ktoe/km^2^, EC: ktoe/km^2^, CO_2_ emission from fossil fuel: kt CO_2_ Equation/km^2^, Fossil-fuel CO_2_ flux: gC m^2^ day^−1^.

Cluster Level	Cluster 1	Cluster 2	Cluster 3	Cluster 4
Decomposed Variables of M Factor	EC: 1.03–1.37	EC: 0.22–0.45	EC: 0.15–0.30	EC: 0.02–0.16
	TEC: 1.34–1.74	TEC: 0.35–0.65	TEC: 0.22–0.47	TEC: 0.06–0.33
	Fossil-fuel CO_2_ flux:	Fossil-fuel CO_2_ flux:	Fossil-fuel CO_2_ flux:	Fossil-fuel CO_2_ flux:
	2.48–3.80	1.10–1.73	0.49–0.77	0.08–0.41
Countries Belonging to the Cluster	BEL, NET	GER, UK, CZE	HUN, AUS, FRA, ITA, POL, SI, SK, SWI	DEN, IRE, BLR, BUL, CRO, EST, FIN, GRE, LAT, LIT, NOR, POR, ROM, SPA, SWE, TUR, UKR
M Factor	M: 0.77–0.79	M: 0.63–0.75	M: 0.59–0.68	M: 0.34–0.75
	Fossil-fuel CO_2_ flux:	Fossil-fuel CO_2_ flux:	Fossil-fuel CO_2_ flux:	Fossil-fuel CO_2_ flux:
	2.48–3.80	1.10–1.73	0.49–0.77	0.08–0.41
Countries Belonging to the Cluster	BEL, NET	GER, UK, CZE	HUN, AUS, FRA, ITA, POL, SI, SK, SWI	DEN, IRE, BLR, BUL, CRO, EST, FIN, GRE, LAT, LIT, NOR, POR, ROM, SPA, SWE, TUR, UKR
Decomposed Variables of E Factor	EC: 1.37	EC: 1.03	EC: 0.22–0.45	EC: 0.02–0.30
	CO_2_: 4.88	CO_2_: 3.22	CO_2_: 1.37–2.29	CO_2_: 0.12–1.10
	Fossil-fuel CO_2_ flux:	Fossil-fuel CO_2_ flux:	Fossil-fuel CO_2_ flux:	Fossil-fuel CO_2_ flux:
	2.48	3.80	1.10–1.73	0.08–0.77
Countries Belonging to the Cluster	NET	BEL	CZE, GER, UK	AUS, BEL, BUL, CRO, DEN, EST, FIN, FRA, GRE, HUN, IRE, ITA, LAT, LIT, NOR, POL, POR, ROM, SI, SK, SPA, SWE, SWI, TUR, UKR
E Factor	E: 13.44	E: 7.11–8.47	E: 4.75–6.59	E: 0.48–4.26
	Fossil-fuel CO_2_ flux:	Fossil-fuel CO_2_ flux:	Fossil-fuel CO_2_ flux:	Fossil-fuel CO_2_ flux:
	0.32	0.25–0.36	0.08–1.73	0.06–3.80
Countries Belonging to the Cluster	EST	BUL, GRE, UKR	BLR, CZE, DEN, FIN, GER, IRE, NOR, POL, POR, ROM, SI, SK, SPA, TUR	AUS, BEL, CRO, FRA, HUN, ITA, LAT, LIT, NET, SWE, SWI, UK
Decomposed Variables of P Factor	Pop: 375.67–507.89	Pop: 205.81–273.05	Pop: 69.78–137.16	Pop: 18.13–46.81
	Fossil-fuel CO_2_ flux:	Fossil-fuel CO_2_ flux:	Fossil-fuel CO_2_ flux:	Fossil-fuel CO_2_ flux:
	2.48–3.80	0.70–1.73	0.25–1.32	0.05–0.32
Countries Belonging to the Cluster	BEL, NET	GER, ITA, SWI, UK	AUS, BUL, CRO, CZE, DEN, FRA, GRE, HUN, IRE, POL, POR, ROM, SI, SK, SPA, TUR, UKR	BLR, EST, FIN, LAT, LIT, NOR, SWE
P Factor	P: 81.10–82.66	P: 60.54–66.87	P: 37.97–46.59	P: 1.32–19.59
	Fossil-fuel CO_2_ flux:	Fossil-fuel CO_2_ flux:	Fossil-fuel CO_2_ flux:	Fossil-fuel CO_2_ flux:
	0.37–1.73	0.58–1.10	0.25–0.76	0.08–3.80
Countries Belonging to the Cluster	GER, TUR	FRA, ITA, UK	POL, SPA, UKR	AUS, BEL, BLR, BUL, CRO, CZE, DEN, EST, FIN, GRE, HUN, IRE, LAT, LIT, NET, NOR, POR, ROM, SI, SK, SWE, SWI
